# Associations of serum C-reactive protein with physical activity, fitness and fatness in South African adolescents

**Published:** 2010-12

**Authors:** H Salome Kruger

**Affiliations:** Centre of Excellence for Nutrition, North-West University, Potchefstroom, South Africa

Globally the prevalence of overweight is increasing significantly, not only in economically developed regions, but also in developing countries. In South Africa, the prevalence of combined overweight and obesity among high-school children increased from 21.2% in 2002^1^ to 25% in 2008,^2^ with 35% of high-school girls being overweight or obese.^2^

The metabolic syndrome (MS) is a cluster of biological markers that together predict the development of cardiovascular disease and type 2 diabetes. These markers are hypertension, insulin resistance, central adiposity, hypertriglyceridaemia and decreased high-density lipoprotein cholesterol (HDL-C). The MS is now increasingly emerging among children and adolescents.^3^ Low-grade systemic inflammation is proposed as a significant component of the MS.^4^

C-reactive protein (CRP) is secreted by the liver and adipose tissue in response to infections and inflammatory conditions.^5^ Levels of CRP decrease during the early convalescent phase and are usually low in healthy subjects. In the absence of infection, low-grade inflammation characterised by serum CRP levels of between 1 and 10 mg/l are associated with an increased risk of the development of cardiovascular disease.^6^ Serum CRP concentrations in this range can be detected using high-sensitivity methods and are generally referred to as high-sensitivity CRP (hsCRP).^6^

Although the MS has been described in paediatric populations from many different countries, limited evidence on the MS and risk for cardiovascular disease among South African children is available.^7^-^9^ Studies on blood lipid levels of South African children without familial hyperlipidaemia are scarce, but published data indicate very little evidence of hypertriglyceridaemia and decreased HDL-C among black South African children.^8^ However, South African studies indicate a positive association between overweight in children and increased blood pressure,^9^ plasminogen activator inhibitor-1 activity, plasma fibrinogen and the thrombin–anti-thrombin complex,^10^ as well as higher fasting plasma insulin possibly increasing the risk for future cardiovascular disease.^7^,^9^

Increased adiposity has been associated with higher serum CRP concentrations in US children aged three to 16 years from the National Health and Nutrition Examination survey (NHANES) 1999–2004.^11^ In a review of research linking obesity and low-grade inflammation in children, a significant positive correlation between body mass index (BMI) and CRP was confirmed.^12^ Ruiz *et al.*^13^ found a significant positive association between body fat, derived from five skin folds, and serum CRP in Swedish children, aged nine to 10 years.

Other variables describing body composition were investigated with regard to their power to predict low-grade inflammation in Caucasian adolescents. Although waist circumference and waist:height ratio showed a significant, positive predictive power to detect elevated serum CRP, BMI was the best predictor of elevated serum CRP levels in these adolescents.^14^ Abdominal obesity in children was also associated with higher serum CRP concentrations in Norwegian children, nine and 15 years old. In the same study, serum CRP was positively associated with blood pressure, blood glucose, insulin and triglyceride concentrations.^15^ In a study of asymptomatic European adolescents, serum hsCRP was also associated with risk for cardiovascular disease. No lifestyle factors showed an association with cardiovascular risk in this study.^16^ These results confirm the value of elevated serum hsCRP as an early marker for cardiovascular disease in older children and adolescents.^15^,^16^

Ridker^17^ reviewed large-scale prospective studies that showed hsCRP is an independent predictor of future cardiovascular events, as well as of hypertension and type 2 diabetes mellitus. Studies in animal models suggest, however, that hsCRP may not promote atherosclerosis directly, but only serve as a marker of vascular inflammation.^18^ Recently Ridker reviewed the evidence from retrospective as well as primary-prevention trials and found that hsCRP was the strongest predictor of risk of vascular events.^19^ A meta-analysis of 54 prospective cohort studies identified and confirmed CRP as an independent risk marker for cardiovascular disease.^20^

A cross-sectional study in Swedish children nine to 10 years old showed a significant negative association between cardiovascular fitness, measured by ergometer bike test, and CRP. No association between physical activity measured by accelerometry and CRP could be found, although physical activity was positively associated with cardiovascular fitness. After controlling for body fat, serum CRP was no longer negatively associated with cardiovascular fitness. The influence of fatness on serum CRP in these children was greater than the influence of fitness.

The results suggest that the beneficial effects of physical activity on low-grade inflammation may be mediated through the association with cardiovascular fitness, but that excessive fatness may decrease the beneficial effects of physical activity in children.^13^ Interventions resulting in about a 5% weight loss in obese children resulted in a decrease in serum CRP concentrations.^12^ Apparently, overweight and obesity in children have a stronger association with serum CRP than physical activity, but physical activity may help to prevent excessive body fat accumulation in children and may even result in moderate fat loss in obese children.

Most studies of body composition, physical activity and lowgrade inflammation in children have been done in Caucasian populations. Longitudinal studies in larger cohorts of different age groups and ethnic backgrounds are needed. Further research is also necessary to assess the changes and effects of other inflammatory mediators, such as tumour necrosis factor alpha (TNF-α), interleukin 6 (IL-6) or IL-8.^12^

In this edition of the *Cardiovascular Journal of Africa*, the results of a study in black South African adolescents indicate a trend of higher serum hsCRP levels in boys with a higher percentage of body fat.^21^ Waist circumference in girls was significantly positively associated with serum hsCRP. In the boys, there was an inverse correlation between percentage body fat and fitness, and between fitness and serum hsCRP, indicating a beneficial effect of physical activity and fitness to decrease their risk for cardiovascular disease. In the girls, a significant difference was found between serum hsCRP levels in the different physical activity categories, with lower serum hsCRP values in girls in the higher physical activity group.

The results indicate that South African children in low-income areas with a low prevalence of obesity are not necessarily protected from an increased risk of cardiovascular disease. Low physical activity has been described among children in such communities.^1^,^2^ Obesity should be prevented in South African children and adolescents by encouraging increased physical activity.
